# New Method of Remedying the Accidents Caused by Chloroform

**Published:** 1851-07

**Authors:** M. Ricord


### ARTICLE XXIII.

### *New Method of Remedying the Accidents Caused by Chloroform.* By M. Ricord.

The following letter, by M. Ricord, has been copied into the *Journal de Chimie*, Jan. 1850, from which I took it, having vainly attempted to get at the original. The subject of which it treats is important enough to merit attention; for few accidents can be more appalling to a surgeon than the death of a patient from the use of a remedy which that surgeon has administered, perhaps much against the wishes of the other.

"M. Ricord," says this Journal, "has just published a very interesting letter, on a method by which he has twice succeeded in rescuing from death two persons whom he brought to the verge of it by the exhibition of chloroform. This method is insufflation by the mouth, without any intermedium; but we will let him speak for himself."

"In my practice I have met with two cases in which the employment of chloroform had nearly been fatal; in both, its action was very rapid, and had not occasioned any previous excitement. It had been administered by means of a sponge, with large pores, which permitted, at least in appearance, the inhalation of a sufficient quantity of air.

"Case 1.—The patient who furnishes the subject of my first case, was a woman of about twenty-six, from whom I was about to remove some growths of no great size. She was previously chloroformed, to which she only submitted after repeated entreaties, for she appeared to be excessively timid.

"The anesthetic effect of the chloroform was very rapid, for after a few respirations she appeared asleep; the sponge was removed, and I commenced excising the growths, but had scarcely given two or three cuts, when one of my assistant-surgeons told me that the pulse appeared to be failing. I now saw, in fact, that the beating of the heart was suspended, that all respiratory movements had ceased, and that the lips were livid, and hung down. The limbs were completely relaxed, and the paleness of the face showed that the patient was in that state of syncope which is the herald of death. All the remedies indicated in such a case were forthwith employed, as cold currents of air, sprinkling cold water on the face, tickling the nostrils, &c. Artificial respiration, by pressure on the walls of the chest, was tried.

"The syncope continued, and death seemed close at hand. I began to be uneasy, and determined to try direct insufflation. I applied my mouth to that of the patient. After some inspirations the dying woman gave a sigh, her chest heaved, the face resumed its normal color, the heart and pulse commenced beating in an appreciable manner, and the eyes opened; respiration had again brought into play all the functions of life, and the return of sensation was evinced by a smile. The patient was saved, and we escaped with the fright.

"Case 2.—The second time that I experienced the dangers of chloroform was with a patient under my care in the Southern Hospital (Hopital du Midi.) He was a young man whose case required circumcision. And this operation was generally painful enough, he asked me to send him to sleep with the chloroform. A sponge, impregnated with it was given him to respire from: the action was very rapid, without any appearance of preceding excitement, and the patient was soon plunged into total insensibility. I performed the operation, but when it was concluded, the patient did not recover his consciousness, and remained in a state of alarming stillness. The pulse gradually sank; the heart ceased to beat; all the sphincters were relaxed, and his cadaverous face seemed to testify that death was near.

"All the means I have indicated in the preceding case were tried, but without avail, and it became necessary to have recourse to direct insufflation, which had already so well succeeded in one case. Success crowned my efforts, and the patient recovered.

"Now, my dear colleague, may we not conclude, from these two instances, that in cases of impending death from the use of chloroform, direct insufflation from mouth to mouth, from the surgeon to the patient, is a more sure and efficacious remedy than anything else ever recommended in such cases; more certain and quicker than all the other methods of artificial respiration with tubes or catheters. Do you not, with me, think that the surgeon who should neglect having recourse to it, would take upon himself a very serious responsibility?

"I know well it will be objected that such a plan is disgusting and repulsive; but this is of very little importance to men whose life is professedly one great act of devotion."

